# Stimulus Phase Locking of Cortical Oscillations for Rhythmic Tone Sequences in Rats

**DOI:** 10.3389/fncir.2017.00002

**Published:** 2017-01-26

**Authors:** Takahiro Noda, Tomoki Amemiya, Tomoyo I. Shiramatsu, Hirokazu Takahashi

**Affiliations:** ^1^Research Center for Advanced Science and Technology, University of TokyoTokyo, Japan; ^2^Institute of Neuroscience, Technical University MunichMunich, Germany; ^3^Graduate School of Information Science and Technology, University of TokyoTokyo, Japan

**Keywords:** regular tone sequences, high-density microelectrode array, auditory cortex, oscillatory phase locking, rat

## Abstract

Humans can rapidly detect regular patterns (i.e., within few cycles) without any special attention to the acoustic environment. This suggests that human sensory systems are equipped with a powerful mechanism for automatically predicting forthcoming stimuli to detect regularity. It has recently been hypothesized that the neural basis of sensory predictions exists for not only what happens (predictive coding) but also when a particular stimulus occurs (predictive timing). Here, we hypothesize that the phases of neural oscillations are critical in predictive timing, and these oscillations are modulated in a band-specific manner when acoustic patterns become predictable, i.e., regular. A high-density microelectrode array (10 × 10 within 4 × 4 mm^2^) was used to characterize spatial patterns of band-specific oscillations when a random-tone sequence was switched to a regular-tone sequence. Increasing the regularity of the tone sequence enhanced phase locking in a band-specific manner, notwithstanding the type of the regular sound pattern. Gamma-band phase locking increased immediately after the transition from random to regular sequences, while beta-band phase locking gradually evolved with time after the transition. The amplitude of the tone-evoked response, in contrast, increased with frequency separation with respect to the prior tone, suggesting that the evoked-response amplitude encodes sequence information on a local scale, i.e., the local order of tones. The phase locking modulation spread widely over the auditory cortex, while the amplitude modulation was confined around the activation foci. Thus, our data suggest that oscillatory phase plays a more important role than amplitude in the neuronal detection of tone sequence regularity, which is closely related to predictive timing. Furthermore, band-specific contributions may support recent theories that gamma oscillations encode bottom-up prediction errors, whereas beta oscillations are involved in top-down prediction.

## Introduction

Repeated, regular sound patterns that are acoustically distinct from noise are commonly observed in animal vocalization, human speech, and natural sound. Thus, detection of regular acoustic patterns is crucial for survival because such patterns probably have behavioral implications. Increasing evidence indicates that animals can inherently distinguish regular sound patterns. For instance, rodents can distinguish tone sequences with different tone orders (Toro and Trobalón, 2005; Murphy and Cook, 2008; Mondragón et al., 2009; Urcelay and Miller, 2010; Perry and Felsen, 2012; de la Mora and Toro, 2013), birds can not only learn rhythmic–arrhythmic discrimination but also distinguish different vocalization patterns with different syntactic rules (Hulse et al., 1984; Shinn-Cunningham et al., 2006; Abe and Watanabe, 2011; Schneider and Woolley, 2013), and primates can recognize and generalize tone patterns beyond specific pitches or stimulus lengths (Ravignani et al., 2013; Wilson et al., 2013, 2015).

Regularity detection in humans is so powerful that regular patterns can be very rapidly detected (i.e., within a few cycles) without any special attention to the acoustic environment (Chait et al., 2008; Jaunmahomed and Chait, 2012). The detection of regular acoustic patterns is accompanied by cortical responses, as measured non-invasively, e.g., transition from a random to a regular pattern is accompanied by an increase in magnetoencephalography (MEG) response (Chait et al., 2008). Neural activity upon the breaking of regular structure is characterized as mismatch negativity (Horváth et al., 2001). Furthermore, late electroencephalography (EEG) components are related to involuntary attention switches (Bendixen et al., 2007; Smith et al., 2010). In addition to these net changes in neural activity, local activity in primary and higher auditory cortical areas is probably involved in the detection of sound regularity and the integration of sequential auditory events (Griffiths et al., 1998; Mustovic et al., 2003). These observations suggest that the human sensory system is equipped with a powerful mechanism for the automatic prediction of forthcoming stimuli, which is used to detect regularity.

The neural mechanisms of sensory prediction have recently emerged a major topic of debate. Recent theories suggest that neural activity can be assessed to detect or predict either what sensory event would occur (predictive coding) or when a particular sensory event would occur (predictive timing) (Friston, 2005, 2009; Wacongne et al., 2011; Arnal and Giraud, 2012; Malmierca et al., 2015). Specifically, cortical gamma-band and beta-band oscillations are differently modulated during beat processing; this suggests that each band is differentially involved in predictive coding or predictive timing (Fujioka et al., 2009, 2015, 2012; Lee et al., 2013; Fries, 2015). Increasing evidence has led to the attractive hypothesis that gamma-band oscillations convey prediction errors regarding both predictive coding and timing in a bottom-up manner, whereas beta-band oscillations convey predictive timing in a top-down manner. For these oscillatory dynamics, entrainment in a specific band (i.e., phase-lock to the structure of the stimulus stream) may be crucial for sensory selection involving predictive timing (Kruglikov and Schiff, 2003; Lakatos et al., 2007; Schroeder et al., 2008) or the build-up effect of auditory or visual objects (Busch et al., 2009; Busch and VanRullen, 2010; Schroeder et al., 2010; Giraud and Poeppel, 2012; Wimber et al., 2012; Noda et al., 2013b; Ossmy et al., 2015; Samaha et al., 2015; Vanvooren et al., 2015; Watrous et al., 2015). These band-specific phase entrainments are probably associated with neuronal detection and/or prediction of regular acoustic patterns. However, this possibility has not been investigated in detail.

We hypothesized that the phases of gamma-band and beta-band oscillations are differentially modulated when sound patterns are predictable or regular, even under anesthesia. This may then serve as the neuronal signature of automatic detection and/or prediction of sound pattern regularity. We densely mapped the auditory cortex of rats under isoflurane-induced anesthesia and determined whether and how band-specific oscillations entrain cortical population activity at the transition from random to regular patterns. We demonstrated that band-specific phase locking changes with sound pattern regularity irrespective of tone patterns, while the evoked response amplitude depends on tone patterns. This evolution of phase locking widely occurred over the auditory cortex irrespective of frequency bands, while modulation of evoked response amplitude was confined around the local foci of activation. These results support our hypothesis that neuronal oscillation in a particular band is critical for the representation of acoustic pattern regularity.

## Materials and methods

This study was conducted in strict accordance with the “Guiding Principles for the Care and Use of Animals in the Field of Physiological Science” published by the Japanese Physiological Society. The experimental protocol was approved by the Committee on the Ethics of Animal Experiments at the Research Center for Advanced Science and Technology at the University of Tokyo (Permit Number: RAC130107). All surgeries were performed under isoflurane-induced anesthesia and animal suffering was minimized.

### Animal preparation

Seven adult male Wistar rats at postnatal week 7 were used in the experiments. Surgeries to expose the auditory cortex were carried out as reported previously (Takahashi et al., 2004, 2005; Shiramatsu et al., 2013). Animals were anesthetized using isoflurane (2.5–3.5% at induction and 1.0–2.0% for maintenance) and held in a fixed position using a custom-made head-holding device. Atropine sulfate (0.1 mg/kg) was administered at the beginning of the surgery and every 7 h to reduce the viscosity of bronchial secretions. A heating blanket was used to maintain body temperature at ~37°C. A skin incision was made at the beginning of the surgery under local anesthesia (1% xylocaine, 0.3 mL). The temporal muscle, cranium, and dura mater overlying the right auditory cortex were surgically removed, and the exposed cortical surface was filled with saline to prevent desiccation. Cerebral edema was minimized by drainage of cisternal cerebrospinal fluid. Respiratory rate, heart rate, and hind paw withdrawal reflexes were monitored to maintain a stable and adequate anesthetic level throughout the recording procedure. Additionally, a small craniotomy was performed near the bregma to embed a reference electrode with an electrical contact to the dura. The ground electrode was placed under the cervical neck skin. The right eardrum was ruptured and waxed to ensure unilateral sound inputs from the ear contralateral to the exposed cortex.

All electrophysiological recordings were performed in a sound-attenuated room (AMC-4015; O'Hara and Co., Ltd., Tokyo, Japan). Microelectrode arrays with 10 × 10 recording site grids and inter-electrode distances of 400 μm (ICS-96 Array; Blackrock Microsystems Inc., Salt Lake City, UT) were used as described previously (Noda et al., 2013b). Neural signals were simultaneously obtained from 96 electrodes (4 corner electrodes were excluded from the analysis). Electrode impedances were approximately 120 kΩ under 1-kHz, 0.1-V sinusoidal waves.

Neural signals were simultaneously amplified with a gain of 1000 and recorded using a neural data acquisition system (Cerebus data acquisition system; Cyberkinetics Inc., Salt Lake City, UT). Local field potentials (LFPs) and multiunit activities (MUAs) were obtained using digital bandpass filters of 0.3–500 Hz and 0.25–7.5 kHz, and sampling frequencies of 1 kHz and 30 kHz, respectively. The spatial distributions of click-evoked LFPs were first mapped on the cortical surface to identify the location of the auditory cortex. The largest focal activation in response to a click was regarded as the center of the anterior auditory cortex (Takahashi et al., 2005) and served as a landmark for the appropriate positioning of the electrode array. The arrays were inserted to a depth of approximately 700 μm below the pial surface to measure LFPs.

### Stimulation

Prior to the main experiments, a frequency response area (FRA) and a characteristic frequency (CF) were identified at each recording site using the MUA, as previously described (Takahashi et al., 2011; Funamizu et al., 2013). Recording sites with a CF were considered parts of the auditory cortex. Only these sites were included in the analyses of the following main experiments. Characterization of the CF is described in detail elsewhere. Briefly, we considered the number of spikes during the 5–45 ms post stimulus onset as a tone-evoked response. The CF at each site was then determined as the frequency at which the test tones evoked a response for the lowest intensity or as the frequency evoking the largest response at a 20-dB SPL (sound pressure level in decibels re 20 μPa), which was the minimum intensity used in this experiment. Multiunit spikes were detected as threshold-crossing events with negative thresholds (−5.65 root mean square [RMS] from the average of the MUA recordings). Test stimuli were tone bursts of 30-ms duration with 5-ms linear rise/fall ramps. The test frequencies ranged between 1.6 and 64 kHz with 1/3-octave increments. Test stimuli had intensities between 20 and 80 dB SPL with 10-dB increments. Since there were 18 test frequencies and 7 intensities used, 126 test tones were used in total. Each tone was presented 20 times in a pseudorandom order with an inter-tone interval of 600 ms.

**Figure 1:**
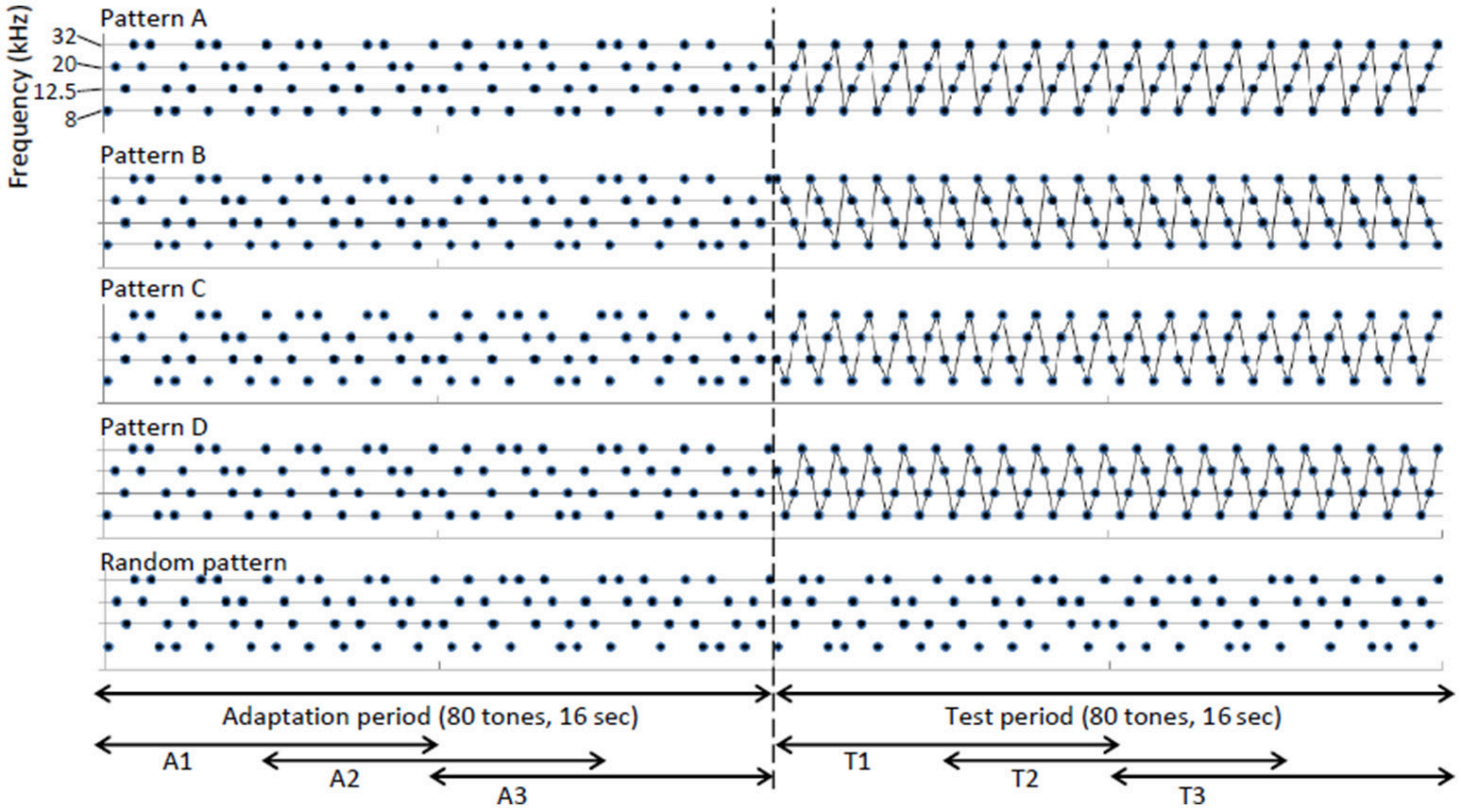
**Design of test stimuli**. Each sequence consisted of 8, 12.5, 20, or 32-kHz element tones with an inter-tone interval of 200 ms. Each tone sequence started with an adaptation period of 16 s (80 tones), which was followed by a 16-s test period (80 tones). During the adaptation period, each element tone appeared in a pseudo-random order. During the test period, a particular order of four element tones was repeated in four test sequences (patterns A–D), while each element tone continued to appear in a pseudo-random order during the control sequence (random pattern). The adaptation and test periods were divided into three sub-periods of 8 s with overlaps of 4 s (A1–A3 and T1–T3).

Figure 1 shows the test stimuli used in the main experiments. Five tone sequences were prepared in total. Patterns A–D were the regular sequences in the main test and the random pattern was used as a control sequence. The inter-sequence interval consisted of 30 s of silence. Each sequence consisted of 8, 12.5, 20, or 32-kHz element tones with inter-tone intervals of 200 ms. All of the tones appeared with equal probabilities (25%). Each element tone was a tone burst of 70 dB SPL and 50-ms duration and had 5-ms linear rise/fall ramps. Each tone sequence started with an adaptation period of 16 s (80 tones), which was followed by a 16-s test period (80 tones). During the adaptation period, each element tone appeared in a pseudo-random order. During the test period, a particular order of 4 element tones was repeated in 4 test sequences (patterns A–D). Each element tone continued to appear in a pseudo-random order in the control sequence (random pattern). To investigate the build-up effect of neural responses, the adaptation and test periods were divided into 3 sub-periods of 8 s with an overlap of 4 s (A1–A3 and T1–T3).

A speaker (EAS-10TH800; Panasonic Corp., Osaka, Japan) positioned 10 cm anterior to the left (contralateral) ear delivered the sounds. Prior to the experiments, all of the frequencies and intensities of the test stimuli were calibrated at the pinna using a microphone (4939; Brüel and Kjær, Nærum, Denmark) and a spectrum analyzer (CF-5210; Ono Sokki Co., Ltd., Kanagawa, Japan). A function generator (WF1974; NF Corp., Kanagawa, Japan) was used to generate test tones for FRA characterization. Tone sequences in the main experiments were generated using a 2-M sample/second 16-bit digital to analog converter (NI USB-6361; National Instruments Corp., Austin, TX) and attenuated by 20 dB using an attenuator (PA5; Tucker & Davis Technologies Inc., Alachua, FL).

### Data analysis

Tone-evoked LFP amplitudes and phase locking were investigated. All data were analyzed using custom-made Matlab codes (Mathworks Inc., Natick, MA).

**Figure 2:**
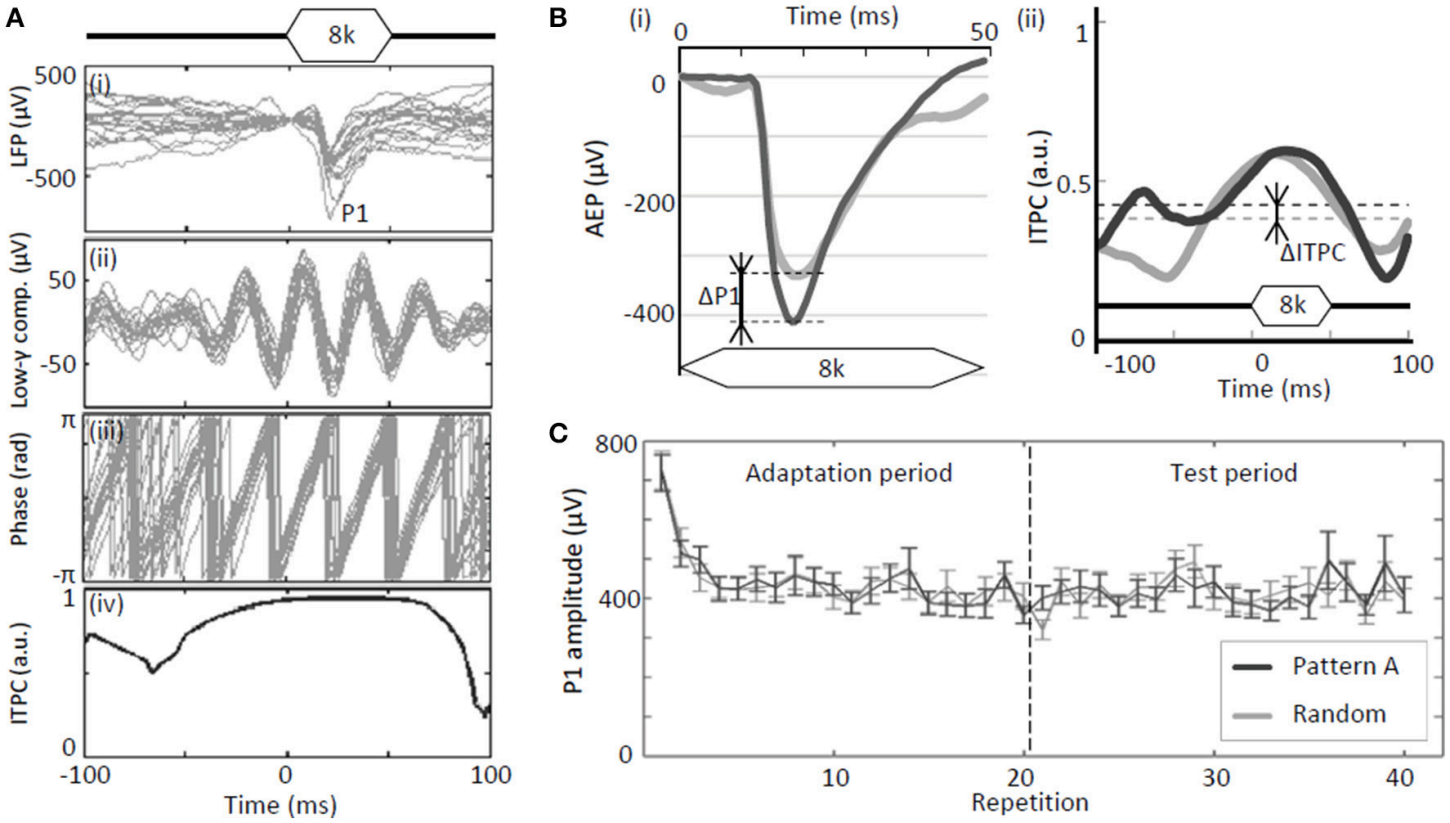
**Test parameters of neural activity. (A)** Analyses of tone-evoked LFPs: **(i)** Raw traces of LFPs; **(ii)** gamma band component; **(iii)** instantaneous phase of gamma band component; and **(iv)** inter-trial phase coherence (ITPC). **(B)** Regular-pattern-induced enhancement of evoked responses (P1) and phase-locking (ITPC): **(i)** ΔP1 and **(ii)** ΔITPC. **(C)** Example of P1 as a function of repetition. A rapid reduction in P1 was observed at the beginning of the adaptation period. Error bars show ±s.e.m.

For each element tone delivered during the test period for a given sequence, the peak amplitudes of evoked LFPs, termed P1 (Figure 2A), were quantified at each site and spatially mapped as functions of test frequency. The differences in the grand-averaged P1s during the test period for each test sequence (patterns A–D) with respect to the control sequence (random pattern) were defined as ΔP1s (Figure 2Bi). Positive ΔP1s indicate that the P1 amplitude of a regular tone sequence is larger than that of a random sequence.

In addition to the P1 amplitudes, phase locking of band-specific LFPs was quantified. The measured LFPs were split into four frequency bands by digital band-pass filtering. These bands are as follows: alpha, 8–14 Hz; beta, 14–30 Hz; low-gamma, 30–40 Hz; and high-gamma, 60–80 Hz. The digital filters used were linear-phase finite-impulse response (FIR) filters, which were designed using the Parks-McClellan algorithm (Matlab; Mathworks Inc., Natick, MA) such that the attenuations at 90% of the low band edge and 110% of the high band edge were 40 dB. The Matlab function “filtfilt” was used to apply a non-causal zero phase filter. Filtered signals were converted into analytic signals of an instantaneous phase using the Hilbert transform (Figure 2A). Phase locking was then quantified using inter-trial phase coherence (ITPC) on the basis of the phase distribution across trials:

$$ITPC\text{​}\left(t\right)=\left|\frac{1}{K}\sum_{k\;=\;1}^{K}exp\left(i\theta \text{​}\left(t_{k}+t\right)\right)\right|$$

where *K* is the number of trials, *t* is the time with respect to the onset of a particular element tone (ranging from −100 to 100 ms in the present analysis), *i* is an imaginary number, θ is the instantaneous phase, and *t*_*k*_ is the time of stimulus onset of a given element tone in the *k*^th^ trial. ITPC is the subtraction of circular variance from 1 (Allen and Johnson, 1991; Marella and Ermentrout, 2008). ITPC ranges between 0 and 1 and nears 1 if the phase at a specific time varies slightly across trials. Otherwise, ITPC is close to 0. The average ITPC values from *t* = −100 ms to *t* = 100 ms were used to evaluate the phase modulation by each tone:

$$\bar{ITPC}=\frac{1}{N}\sum_{ch}\left\{\frac{1}{T}\sum_{t}ITPC\text{​}\left(t\right)\right\}$$

Similar to P1, the differences between $\bar{ITPC}$ during the test period for each regular sequence (patterns A–D) and $\bar{ITPC}$ in the control sequence (random pattern) were defined as ΔITPC (Figure 2Bii). A positive ΔITPC indicates that a regular tone sequence leads to stronger phase locking than a random sequence.

To evaluate the spatial spread of the regularity-induced modulation of P1 amplitude and ITPC, ΔP1 and ΔITPC at each site were quantified as a function of distance from a P1 local focus based on either a physical distance (mm) or a tonotopic distance (octave). Tonotopic distance was defined as the difference in CF between a pair of given sites.

To test the significance of regularity-induced modulation, two-way analysis of variance (ANOVA) was used considering the following null hypotheses: (i) ΔITPCs are equal between the adaptation and test periods (main-period factor; A1–A3 vs. T1–T3) and that (ii) ΔITPCs are equal among the sub-periods (sub-period factor) either during the adaptation periods (A1 vs. A2 vs. A3) or the test periods (T1 vs. T2 vs. T3). The main-period factor tested whether a transition from random to regular tone sequences modulated ΔITPC. If so, the sub-period factor tested whether ΔITPC evolved over time.

## Results

For both regular-tone and random-tone sequences, P1 amplitudes of tone-evoked responses rapidly decayed during the initial several seconds owing to adaptation to the tones; however, it relatively stabilized thereafter (Figure 2C). Therefore, our major interests are the P1s and ITPCs of these fully adapted, stable responses during the test period, i.e., repetitions 21–40 (8–16 s after the onset of the tone sequences).

### Tonotopic representations of P1 and ITPC

**Figure 3:**
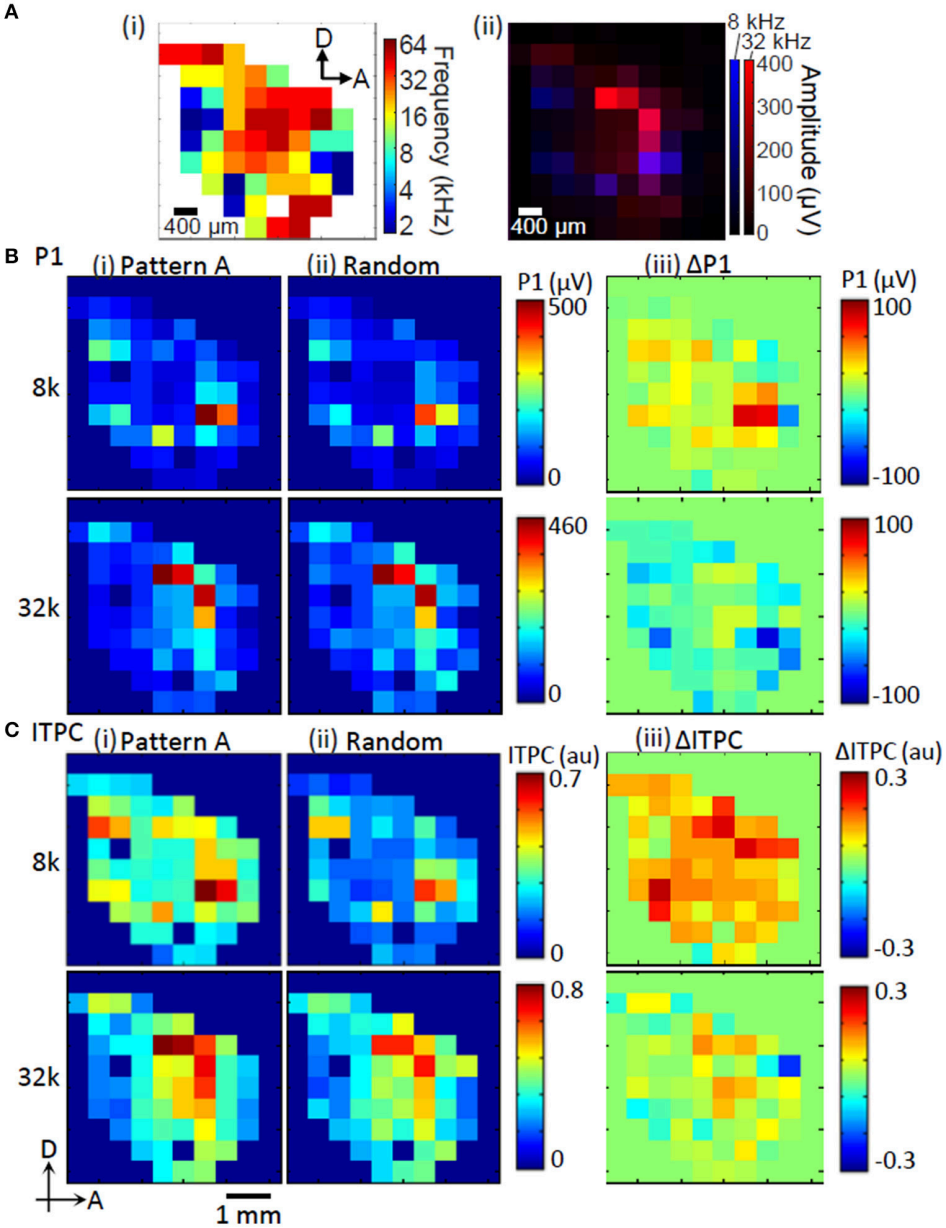
**Representative map of tone-evoked response amplitudes and phase-locking. (A)** Tonotopic map obtained from MUA **(i)**. Tonotopic activation of P1 **(ii)**. **(B)** P1 amplitude. **(C)** ITPC at low gamma band. **(i)** Regular sequence (Pattern A); **(ii)** random sequence; and **(iii)** enhancement of P1 and ITPC during regular sequences compared to those random sequences (ΔP1 and ΔITPC). The test frequencies used to examine these maps were 8 and 32 kHz.

The test region exhibited a clear tonotopic map, or a place code for frequency. These maps are routinely characterized using MUAs (Figure 3Ai). The primary auditory cortex (AI) in the most dorsal auditory field contains a complete high-to-low tonotopic gradient running along the rostral-to-caudal axis. The anterior auditory field (AAF) has a complete high-to-low tonotopic gradient along the posterodorsal-to-anteroventral axis. The ventral auditory field (VAF) and the suprarhinal auditory field (SRAF) about the ventral border of the AI and the posterior border of the AAF. The posterior auditory field (PAF) is defined as posterior to the A1. Finally, the anterior ventral auditory field (AVAF) is defined on the basis of tonotopic discontinuity at the ventral border of the AAF and the anteroventral border of the SRAF (Horikawa et al., 1988; Rutkowski et al., 2003; Kalatsky et al., 2005; Polley et al., 2007; Higgins et al., 2010; Storace et al., 2011; Takahashi et al., 2011; Funamizu et al., 2013). Consistent with this map, the P1 exhibited tonotopic representation, where a low-frequency tone (8 kHz) led to several activation foci appearing at the fringe, while a high-frequency tone (32 kHz) appeared in the middle (Figure 3Aii).

The tonotopic patterns of P1 were qualitatively similar during the regular and random sequences (Figures 3Bi,ii). However, the differential patterns between regular and random sequences, i.e., ΔP1, revealed that ΔP1 was dependent on the tones of the test elements. Here, ΔP1 tended to be positive in response to 8-kHz tones and was negative in response to 32-kHz tones (Figure 3Biii). ITPC was highly correlated with P1 (Pearson's coefficients between P1 and ITPC map: 8 kHz, *R* = 0.83; 32 kHz, *R* = 0.81; Figures 3B,C). This indicates that ITPC is also tonotopically organized. The differential patterns of ITPC in response to regular and random sequences, i.e., ΔITPC, were also dependent on the tones of the test elements.

### Neural signature of regularity

**Figure 4:**
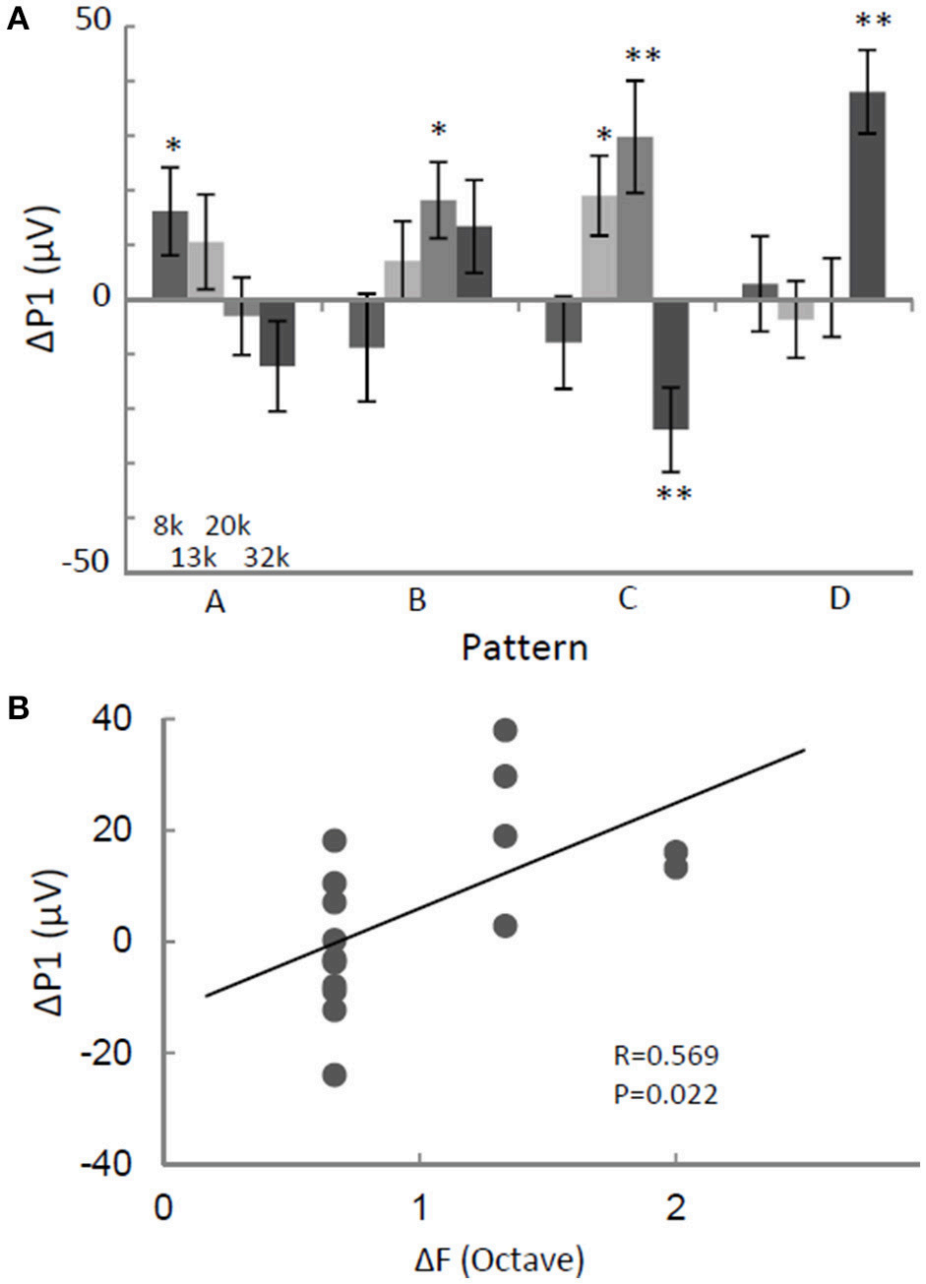
**ΔP1 depends on the test stimulus. (A)** ΔP1 as a function of sequence pattern. P1 in response to an identical tone depended on the sequence patterns. **(B)** ΔP1 as a function of frequency separation with respect to the preceding tone. ΔP1 of the largest P1 in the activation map was quantified for the presented tone. Two-sided *t*-test, ^*^p < 0.05; ^**^p < 0.01. Error bars show ±s.e.m.

We investigated whether the regularity of a tone sequence modulated P1 independently of the test pattern. The ΔP1 of the largest P1 in the activation map was quantified as a function of the test stimuli. For identical tones, ΔP1 depended on test patterns. It was significantly positive in response to some patterns, but was negative to in response to others (two-sided *t*-test, *p* < 0.05; Figure 4A). This pattern-dependent ΔP1 was likely caused by forward masking because ΔP1 was significantly positively correlated with the separations between the provided test frequencies and those of the preceding tones (ΔF) (Figure 4B). In other words, the P1 in response to a given tone was suppressed when a subset of the neural population preferring the test tone was activated by the preceding tone with an adjacent frequency. This result suggests that P1 amplitude is not a candidate for the pattern-independent neural representation of regular tone sequences.

**Figure 5:**
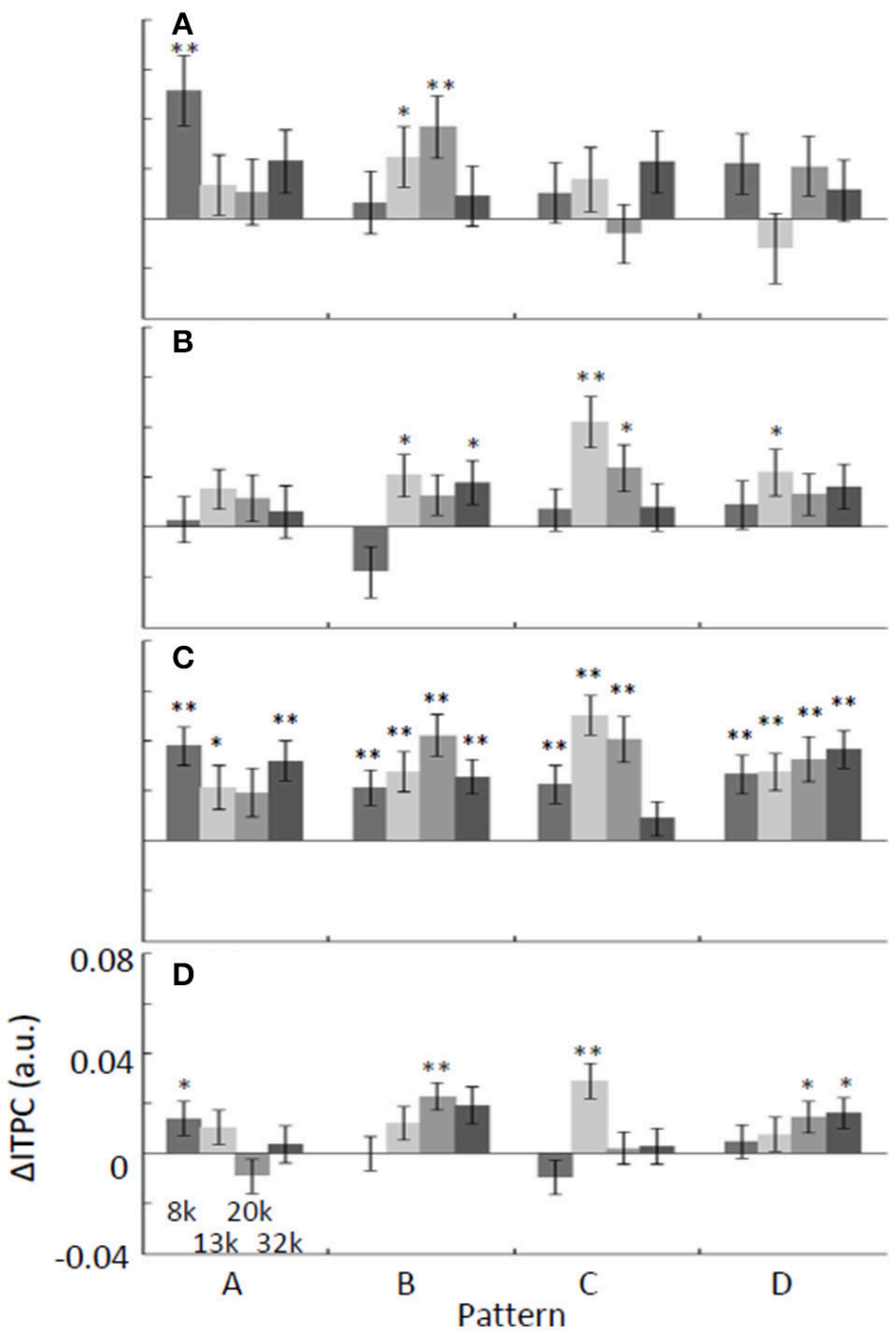
**Band-specific ΔITPC depends on the test stimulus: (A)** alpha, **(B)** beta, **(C)** low-gamma, and **(D)** high-gamma. The average of ΔITPC over the auditory cortex was quantified. ΔITPC tended to be positive regardless of the pattern and frequency of the tone, especially for low-gamma oscillations. ^*^p < 0.05; ^**^p < 0.01.

**Figure 6:**
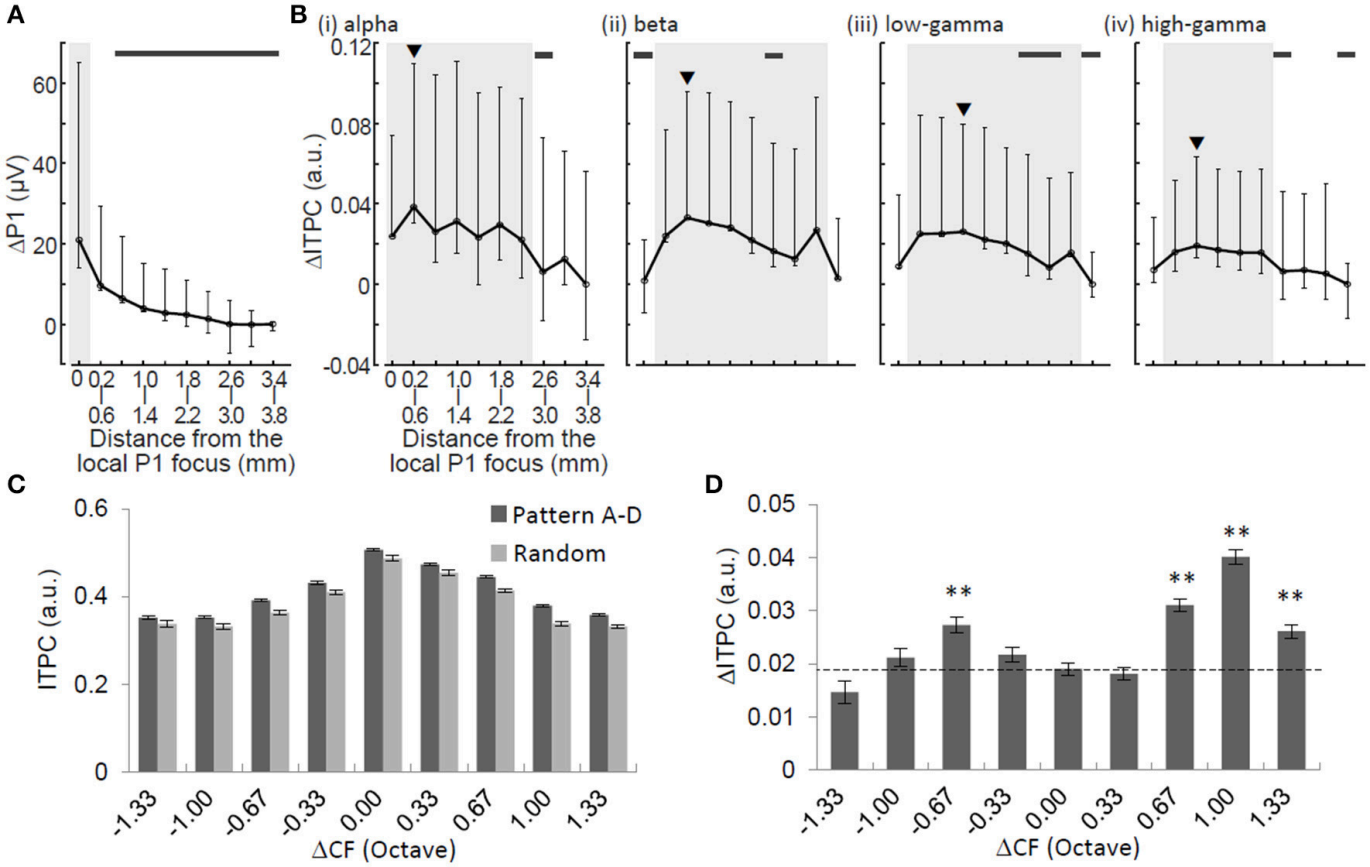
**Spatial characterizations of ΔP1 and ΔITPC**. ΔP1 **(A)** and ΔITPC **(B)** as functions of physical distance from the P1 local focus. Median and error bars indicating 25 and 75% quartile deviations are provided. Black arrowheads in **(B)** indicate the local maxima of ΔITPC. Shaded areas indicate the ranges of half-widths in relation to the local maxima. Thick horizontal bars indicate that ΔP1 or ΔITPC at a given distance are significantly smaller than the local maxima (Mann-Whitney *U*-test with Bonferroni correction, *p* < 0.05). ITPC **(C)** and ΔITPC **(D)** in the low-gamma band as a function of tonotopic distance (ΔCF in octave). Two-sided *t*-test, ^**^p < 0.01.

We also tested whether ΔITPC at a given frequency band may be the neural correlate for the extraction of regular tone sequences (Figure 5). The average of ΔITPC over the auditory cortex was quantified, as ITPC was less localized in the auditory cortex than P1 was (Figures 3, 6). Similar to ΔP1, ΔITPC in response to identical tones depended on the test patterns. However, we found pattern-independent modulation in the low-gamma band, where almost all of the test tones were significantly positive (two-sided *t*-test, *p* < 0.05; Figure 5C). This suggests that the low-gamma ITPC increases in the presence of any kind of regular tone sequence.

### Spatial spread of neural signature

To characterize the spatial spread of the neural signature of regularity, we compared ΔP1 and ΔITPC in terms of spatial profile. ΔP1 exhibited local maxima around the activation foci of P1 and monotonically decreased with distance from the focus with a halfwidth of <0.4 mm (Figure 6A). However, ΔITPC did not have a local maximum around the P1 focus. It had a non-monotonic function with respect to the distance from the foci of the local maxima depending on the oscillation band (Figure 6B): alpha, 0.2–0.6 mm (with a halfwidth of <2.4 mm); beta, 0.6–1.0 mm (halfwidth, <2.8 mm); low-gamma, 1.0–1.4 mm (halfwidth, <2.4 mm); and high-gamma, 0.6–1.0 mm (halfwidth, <1.6 mm). The spatial decays of ΔITPC, where ΔITPC became significantly smaller than the maximum ΔITPC, were 2.2–2.6 mm in the alpha band, 1.4–1.8 mm in the beta band, 1.0–1.4 mm in the low-gamma band, and 1.4–1.8 mm in the high-gamma band. These spatial decays were significantly larger than those of ΔP1 (0.6–1.0 mm) (Mann-Whitney *U*-test with Bonferroni correction, *p* < 0.05). These results suggest that ΔITPC spreads more widely over the auditory cortex than ΔP1.

We further characterized the spatial spread of low-gamma ITPC and ΔITPC, because these phase-locking features are candidates for the neural signature representing sound pattern regularity (Figure 5). The low-gamma ITPC significantly depended on the separation of the test frequency from the CF at a given recording site (i.e., tonotopic distance or ΔCF) during both the adaptation and test periods [Figure 6C; one-way ANOVA: *F*_(8, 31, 796)_ = 405.38, *p* < 10^−4^ for the random sequence; *F*_(8, 7975)_ = 136.21, *p* < 10^−4^ for the regular sequence]. Consistent with the spatially averaged data (Figure 5), the low-gamma ITPCs in the regular patterns were higher than that in the random patterns at any site in the auditory cortex, i.e., irrespective of ΔCF. Closer inspection revealed that the ΔITPCs at the CF sites of the test tones (ΔCF = 0) were significantly lower than those at the surrounding sites [Figure 6D; ΔCF approximately ± 0.67 octave; one-way ANOVA, *F*_(8, 31, 796)_ = 33.3, *p* < 10^−4^; *post-hoc* two-sided *t*-test, *p* < 0.01]. This suggests that tone repetition exerts a more considerable impact on phase locking at frequencies surrounding the CF rather than the CFs of the test tones.

### Evolution of phase locking modulation

**Figure 7:**
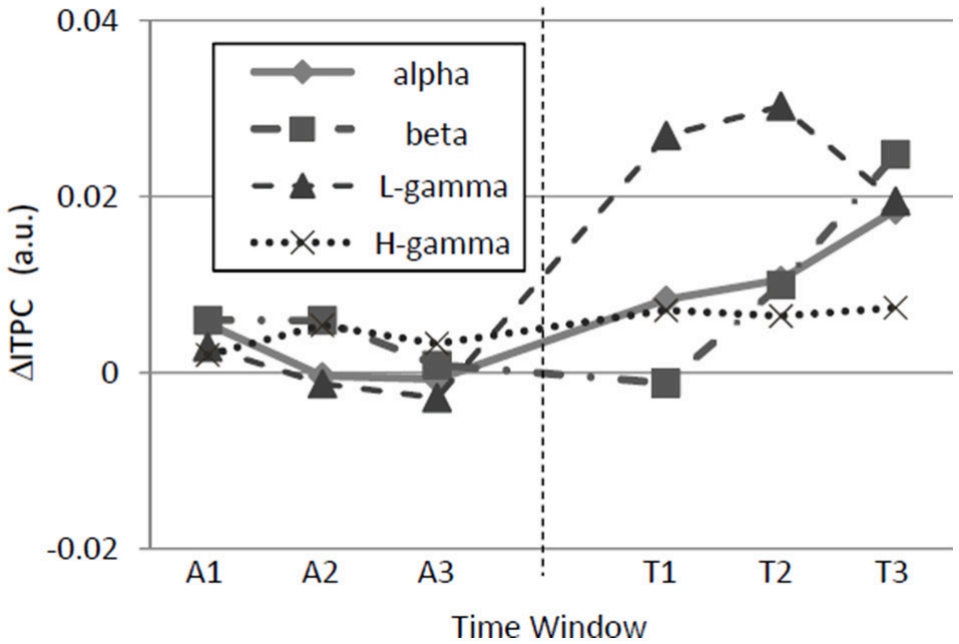
**Time variations of band-specific ΔITPCs**. In the alpha, beta, low-gamma, and high-gamma bands, ΔITPC was significantly higher during the test period than during the adaptation period. Additionally, in the beta band, ΔITPC evolved over time during the test period. Similar to Figure 5, ΔITPC is the averaged value across the auditory cortex.

The neuronal signature of regularity might be produced in either a bottom-up or a top-down manner. A bottom-up signature probably evolves immediately upon the switch from a random to a regular sequence, whereas a top-down signature probably evolves gradually with time after the switch. Thus, we investigated the time courses of ΔITPC and found that ΔITPC evolves in a band-specific manner (Figure 7). Significant main-period effects were observed in the alpha [*F*_(1, 90)_ = 12.92, *p* = 0.00052] and low-gamma [*F*_(1, 90)_ = 103.26, *p* < 10^−4^] bands without sub-period effects or interactions between the two factors. More interestingly, the beta band ΔITPC exhibited a significant main-period effect [*F*_(1, 90)_ = 8.2, *p* = 0.0052], a sub-period effect [*F*_(2, 90)_ = 6.21, *p* = 0.0030], and an interaction between these two factors [*F*_(2, 90)_ = 14.07, *p* < 10^−4^]. The sub-period effect in the beta band was significant only during the test period [one-way ANOVA, *F*_(2, 45)_ = 13.89, *p* < 10^−4^]. No significant effect was observed in the high-gamma band. Along with the results shown in Figure 5, regular-tone sequences tended to induce phase locking in the alpha, beta, and low-gamma bands. The phase locking in the beta band evolved with time.

### Amplitude modulation vs. phase locking modulation

**Figure 8:**
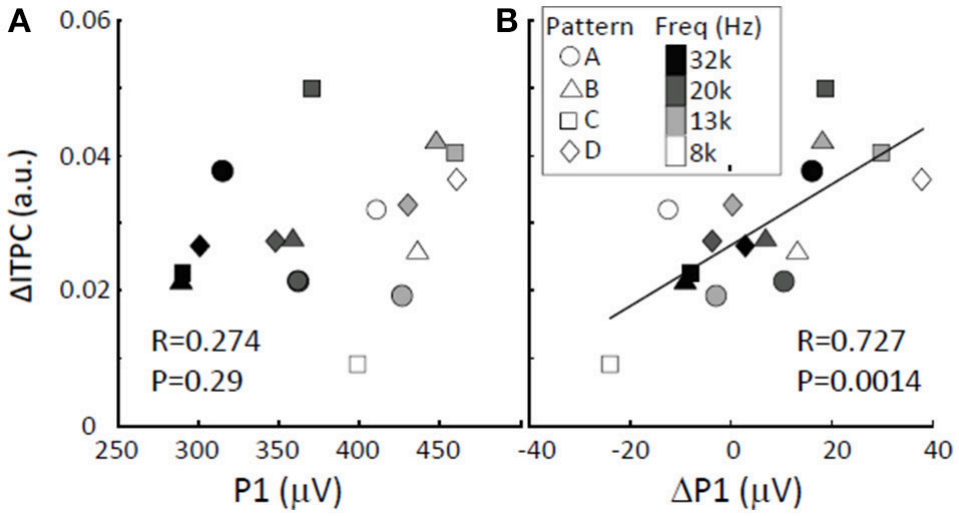
**ΔITPC vs. LFP amplitude. (A)** No correlation was found between low-gamma ΔITPC and P1. **(B)** Positive correlation between low-gamma ΔITPC and ΔP1. The largest P1 and the corresponding ΔP1 were obtained from the activation map in response to the presented tone, while the ΔITPC is the averaged value across the auditory cortex.

We determined whether and how low-gamma ΔITPC depended on the amplitudes of tone-evoked responses. There was no significant correlation between low-gamma ΔITPC and P1 (Figure 8; Pearson's coefficient *R*_*xy*_ = 0.274; two-sided *t*-test, *p* = 0.29). This indicates that large auditory evoked responses do not lead to increased phase locking upon the transition from random to regular tone sequences. However, low-gamma ΔITPC was significantly positively correlated with ΔP1 (Pearson's coefficient *R*_*xy*_ = 0.727; two-sided *t*-test, *p* = 0.0014). This suggests that increases in P1 in response to particular tones trigger tight phase locking upon the transition from a random to a regular sequence and that they enhance overall phase locking to tones in response to repeated sequences.

## Discussion

### Summary of findings

Neuronal responses specific to regular tone sequences in the auditory cortex were characterized by comparing tone-evoked responses to regular sequences with those in responses to random sequences. The amplitudes of tone-evoked responses (P1) depended on test patterns such that P1 increased when the frequency separation between a given tone and the prior tone was large during regular sequences (Figure 4). Therefore, P1 amplitude is likely to encode the local order of the test patterns rather than serve as a global sign of sound repetition. In contrast to P1 amplitude, band-specific phase locking to tones (ITPC) tended to be larger during regular tone sequences than during the random sequences (Figure 5). This enhancement in phase-locking (ΔITPC) was observed widely over the whole auditory cortex area (Figures 3, 6). These results support the idea that widespread phase locking to regular tones plays a more important role in the neural processes involved in detecting regularity of tone sequences rather than those responsible for the detection of the local order of tone sequences. We found that phase locking evolves in a band-specific manner. Gamma-band phase locking increases immediately after the transition from random to regular sequences, while beta-band phase locking gradually evolves with time after the transition (Figure 7). Thus, each band is likely to play a different role in the neuronal detection of repeated tone sequences at different points of temporal evolution. We also observed that pattern-dependent enhancements of phase locking were positively correlated with those of P1. This suggests that increases in P1 in response to particular tones trigger tight phase locking (Figure 8).

### P1 amplitude vs. ITPC

P1, which is a major short latency response component, exhibited rapid adaptation to repeated tone sequences (Figure 2C). Since the recording sites were located at the depth of the thalamo-recipient layer in the cortex, the major mechanisms of this adaptation might involve fatiguing of thalamic inputs (Antunes and Malmierca, 2011, 2014) and thalamo-cortical synaptic depression (Wehr and Zador, 2005; Taaseh et al., 2011; Hershenhoren et al., 2014; Nelken, 2014). The degree of depression depends on the frequency difference between the present tone and the preceding tone. This is known as forward masking (Brosch and Schreiner, 1997; Noda et al., 2013b).

Stimulus phase locking is partly caused by highly reliable tone-evoked responses (Edwards et al., 2009). In fact, the spatial distributions of ITPC were tonotopically organized (Figure 3C), similar to the P1 distributions. The increments of ITPC in response to a regular tone sequence compared to a random tone sequence also showed tight positive correlations with those of the P1 amplitude (Figure 8).

We characterized the P1 amplitude as the average of the peak LFP values in each trial. Therefore, instead of the peak of the trial-averaged evoked response, P1 amplitude is theoretically independent of temporal and phase information in a sharp LFP transient. The time window to quantify phase-locking (200 ms) was also much longer than the duration of the P1 component (several tens of milliseconds). Thus, ITPC is essentially different from P1 amplitude. These test parameters differ in several respects. First, the spatial distributions of the ITPC increment were less local and were more widely distributed than those of P1 amplitude. Second, the increments of ITPC were always positive even though no P1 increment was observed in some test conditions. Third, the coefficient of variation of the P1 amplitude in response to various tone patterns was not correlated with the corresponding ITPC. This indicates that amplitude differed from phase in terms of inter-trial variability (Supplementary Figure 1). Fourth, the regularity-induced evolution was observed only in phase locking but not in P1 amplitude. Fifth, a rapid fall-off was clearly observed in P1 amplitude within a few repetitions of the tones. However, there was no such fall-off in ITPC (Noda et al., 2013a). The differences between P1 amplitude and ITPC are likely caused by the phase reset of the ongoing LFP without evoking P1 responses (Makeig et al., 2002; Sauseng et al., 2007; Barry, 2009; Kayser, 2009; Lakatos et al., 2009).

### Spatial spread of phase locking

The wide distribution of phase-locking compared to P1 amplitude suggests that the neural mechanism of phase locking (e.g., phase reset) works with a low level of thalamo-cortical input, while the local distribution of the P1 amplitude is shaped by feedforward side-band inhibition (Wang and Salvi, 2002; Zhang et al., 2003; Llinás et al., 2005). Additionally, phase locking may be enhanced over a wide range by cortico-cortical horizontal connections via the axonal collaterals of pyramidal cells or interneurons. This may lead to synchronization of neuronal populations and the transmission of signals in a band-specific manner (Traub et al., 1996; Buzsáki and Wang, 2012). Stable global oscillations with high phase locking are established in the presence of regular tone sequences because a number of oscillatory activities are simultaneously enabled by reliable thalamo-cortical inputs.

An increase in ITPC in response to regular tone sequences was prominent in CF regions 0.67–1.00 octave away from regions with the CF of the test frequency (Figure 6D). This spatial spread of phase locking may be enabled by short-range intracortical connections (Happel et al., 2010) and contributes to the grouping of tones (e.g., 8–12.5–20 kHz and 12.5–20–32 kHz). From a computational perspective, such grouping through temporal phase-locking makes the encoding of regular patterns more robust than that of random patterns without consuming additional energy (Buzsaki and Draguhn, 2004; Fell and Axmacher, 2011; Yokota et al., 2015).

### Band-specific phase locking

We found that gamma-band phase locking immediately increases in the first sub-period (0–8 s) of the regular tone sequence and maintains a high value throughout the remaining sub-periods of the sequence (4–12 and 8–16 s) (Figure 7). However, beta band phase locking gradually increases during the second sub-period (4–12 s) and reaches its maximum during the last sub-period (8–16 s). These band-specific temporal evolutions suggest that each band plays a different role in our experiments.

Gamma oscillations have long been known to play a role in the temporal binding of sensory features into a coherent percept (Gray et al., 1989; Engel et al., 2001). According to this traditional viewpoint, the gamma oscillations present during the presentation of regular tone sequences in our study may indicate that repeated tones are temporally bound as an auditory object. Additionally, the rapid evolution of gamma oscillations suggests that this binding process is an automatic bottom-up process that is a “primitive” auditory feature. This gamma band synchronization is likely to occur locally in a feedforward manner.

Compared to the gamma rhythm, the slower rhythms in the alpha to beta bands are used to organize longer-range spatial and temporal synchronous activity (Kopell et al., 2000; Siegel et al., 2012; Lee et al., 2013; Fries, 2015). For example, beta band modulation is associated with motor control not only in the cortex (Engel and Fries, 2010) but also in the thalamus (Paradiso et al., 2004) and basal ganglia (Cassidy et al., 2002; Foffani et al., 2005). In addition, beta activity in the auditory cortex is associated with beat processing in concert with the motor and association cortices and the cerebellum (Fujioka et al., 2012, 2015). The beta band activities in the cortex emerge from the deep cortical layers (Wang, 2010; Markov et al., 2013) and may transmit signals in a feedback or top-down manner (von Stein and Sarnthein, 2000; Buschman and Miller, 2007; Chandrasekaran and Ghazanfar, 2009; de Graaf et al., 2013).

Gamma-band and beta-band phase locking may be differentially involved in predictive timing. According to the predictive coding hypothesis, gamma oscillations convey bottom-up prediction errors in sensory information (e.g., violation of sound expectation), whereas beta oscillations provide top-down prediction (Friston, 2005, 2009; Arnal and Giraud, 2012; Siegel et al., 2012; Malmierca et al., 2015). During the random sequence, where the prediction of the upcoming tone is impossible, no prediction error can be produced. Therefore, there is no predictive timing during the random sequence. Immediately after the transition from the random to the regular sequence, temporal regularity should enable predictive coding, possibly with a transition response (i.e., the largest error signal) appearing within a few cycles after the transition (Chait et al., 2008). Such immediate initiation of predictive coding should simultaneously enable predictive timing, which is associated with the rapid evolution of gamma-band phase locking in this present study. Thereafter, stable prediction may then become gradually available, as indexed by the gradual evolution of beta-band phase locking. Thus, our results may provide additional evidence that gamma and beta-band phase locking play compensatory roles in predictive timing.

Our results suggest that predictive timing is active under anesthesia. We have characterized phase locking in an early component of evoked potential, which is less vulnerable to conscious states than late response components (Del Cul et al., 2007). There are also several pre-attentive mechanisms in predictive coding. These include stimulus-specific adaptation and mismatch negativity (Friston, 2005; Peretz et al., 2009; Winkler et al., 2009; Wacongne et al., 2011, 2012; Bendixen et al., 2012; Shiramatsu et al., 2013; Malmierca et al., 2015). However, further experiments are still required to elucidate the effects of anesthesia on our findings, as isoflurane has substantial impacts on oscillatory activities (Imas et al., 2005; Noda and Takahashi, 2015). Anesthesia also induces loss of consciousness (Alkire et al., 2008; Raz et al., 2014), which weakens top-down information transfer. The powerful top-down control exerted by attention during waking may alter the neural signature of regularity. Awake-state behavioral experiments will promote a better understanding of how top-down and bottom-up mechanisms contribute to the perception of tone sequence regularity. In these experiments, lengthening the duration between the tones would allow us to investigate whether tone sequence regularity modulates tone-induced oscillatory components (Tallon-Baudry et al., 2004; Tallon-Baudry and Bertrand, 2007). It would be also intriguing to comprehensively characterize tone pattern-specific phase locking across different frequency bands. Furthermore, causal links between neural activity in the auditory cortex and other regions is of great interest to us and can be used to address whether and how beta and gamma phase locking are associated with top-down and bottom-up information flow, respectively.

In summary, regular-tone sequences spatio-temporally modulate the stimulus phase locking of neural oscillations in the auditory cortex with global scales, whereas the amplitudes of tone-evoked responses to each component encode the sequence information with local scales, i.e., the local order of tones. Additionally, band-specific temporal evolution of oscillatory phase locking may support the recent theories regarding predictive timing that gamma-band phase locking encodes bottom-up prediction errors while beta-band phase locking is involved in top-down prediction.

## Author contributions

TN, TA, and HT designed experiments. TN, TA, and HT analyzed data. TN, TA, and TS conducted physiological experiments. TN, TA, and HT evaluated results. TN, TA, and HT made all figures. TN, TA, and HT wrote the manuscript.

## Funding

This work was partially supported by KAKENHI Grant (25135710, 26242040, 16H01604).

### Conflict of interest statement

The authors declare that the research was conducted in the absence of any commercial or financial relationships that could be construed as a potential conflict of interest.
