# Supplementary material for: Stimulus Phase Locking of Cortical Oscillations for Rhythmic Tone Sequences in Rats
Source: Front Neural Circuits. 2017 Jan 26;11:2. doi: 10.3389/fncir.2017.00002 (PMC5266736; doi:10.3389/fncir.2017.00002)
Supplement: Supplementary file 1 [file Image1.PDF]

Supplementary Material

Stimulus phase locking of cortical oscillation for rhythmic tone sequence in rats

Takahiro Noda[1, 2†], Tomoki Amemiya[3†], Tomoyo Isoguchi Shiramatsu[1], Hirokazu Takahashi[1,3*]

*** Correspondence:** Hirokazu Takahashi: takahashi@i.u-tokyo.ac.jp

1 Supplementary Data

In order to compare the variability of P1 amplitude with the inter-trial phase coherence i.e., the variability of phase, coefficient of variation (CV) of P1 amplitude was quantified across trials:

$$\boldsymbol{CV = \sigma(P1)/\overline{P1}}$$

where *P1* is the P1 amplitude in each trial, $\sigma(P1)$ is standard deviation of the P1 amplitude. The larger CV is, more variable the P1 amplitude across trials is. The difference of CV, ΔCV was evaluated by subtracting the CV in the control (random) sequence from the CV in each regular sequence during the test period.

Supplementary Figure 1 shows the ΔCVs for different tone sequence patterns. Significantly negative ΔCVs, or more reliable P1 amplitudes were observed only at pattern A and D conditions (two-sided t-test, *, $p<0.05$). Note that the profile of ΔCV was not similar to any profile of band-specific ΔITPC in Fig. 5.

2 Supplementary Figures and Tables

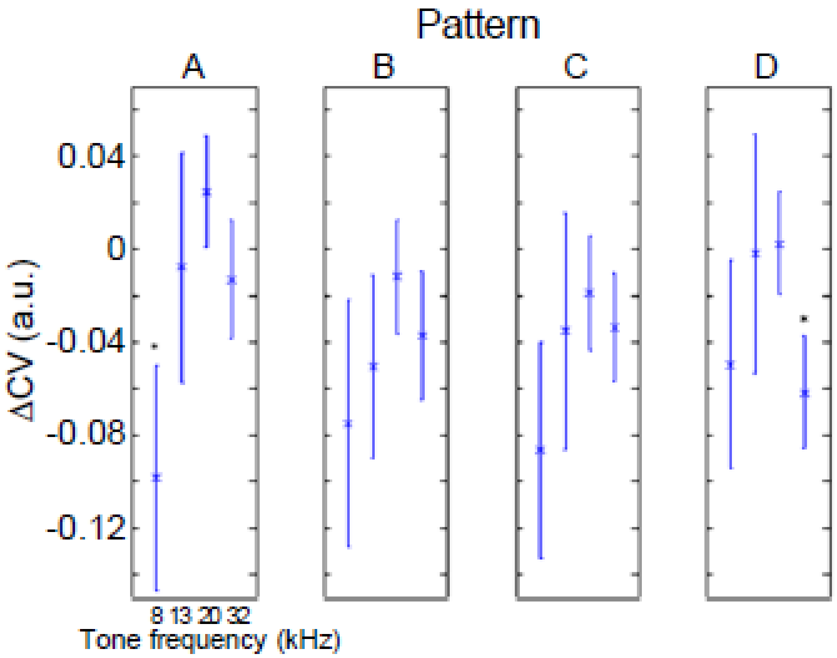

Supplementary Figure 1. The tone-pattern dependent ΔCV. Significant reduction of CV was observed at pattern A and D conditions. Error bars show SE (n = 70). The behavior of ΔCV across various tone patterns was different from that of any band-specific ΔITPC in Fig. 5.
